# Velo-Predictor: an ensemble learning pipeline for RNA velocity prediction

**DOI:** 10.1186/s12859-021-04330-1

**Published:** 2021-09-03

**Authors:** Xin Wang, Jie Zheng

**Affiliations:** School of Information Science and Technology, ShanghaiTech University, 393 Middle Huaxia Road, Pudong District, 201210 Shanghai, China

**Keywords:** RNA velocity, Single cell, Ensemble learning, Landscape

## Abstract

**Background:**

RNA velocity is a novel and powerful concept which enables the inference of dynamical cell state changes from seemingly static single-cell RNA sequencing (scRNA-seq) data. However, accurate estimation of RNA velocity is still a challenging problem, and the underlying kinetic mechanisms of transcriptional and splicing regulations are not fully clear. Moreover, scRNA-seq data tend to be sparse compared with possible cell states, and a given dataset of estimated RNA velocities needs imputation for some cell states not yet covered.

**Results:**

We formulate RNA velocity prediction as a supervised learning problem of classification for the first time, where a cell state space is divided into equal-sized segments by directions as classes, and the estimated RNA velocity vectors are considered as ground truth. We propose Velo-Predictor, an ensemble learning pipeline for predicting RNA velocities from scRNA-seq data. We test different models on two real datasets, Velo-Predictor exhibits good performance, especially when XGBoost was used as the base predictor. Parameter analysis and visualization also show that the method is robust and able to make biologically meaningful predictions.

**Conclusion:**

The accurate result shows that Velo-Predictor can effectively simplify the procedure by learning a predictive model from gene expression data, which could help to construct a continous landscape and give biologists an intuitive picture about the trend of cellular dynamics.

## Background

Recent advances in high-throughput RNA sequencing technologies [1] have enabled analysis of transcription at single-cell level [2], which has provided immense opportunities to unravel the underlying mechanisms of gene expression regulation. However, in many cases, dynamical information of cell state transition is limited. When the sequencing is completed, the expression data provide only a snapshot of a cell [3]. Currently, trajectory inference (including pseudotime analysis) is a primary task to identify cells in various states of differentiation [4]. In general, trajectory inference methods need to construct graphs. There are various approaches to trajectory reconstrcution, e.g. SCUBA [5] is based on bifurcation analysis, SCENT [6] and scEpath [7] use a measurement of entropy of cell states. HopLand [8] and Topslam [9] project cells to a landscape with optimized parameters.

A major limitation of most trajectory inference methods [10] is they do not connect data to underlying molecular kinetics. La Manno et al. found that spliced and unspliced mRNAs can be distinguished in standard single-cell RNA-seq protocols [11], and the timescale of differentiation during development is comparable to the typical half-life of an mRNA. Hence, we can use the abundances of mRNAs to estimate splicing rate and degradation rate. They proposed a simple kinetic framework for estimating changes in mRNA levels of individual cells. This framework is based on the central dogma of molecular biology. Gorini and Maas proposed a first-order differential equation to model this biological process [12], to which Zeisel et al. added intermediate steps [13].

The original steady-state model for RNA velocity proposed by La Manno et al. assumes that transcriptional phases endure long enough to reach a steady state equilibrium, and the equilibrium mRNA levels can be approximated with a linear regression by simplification with a common splicing rate. Recently, to relax this assumption, Volker Bergen et al. proposed an algorithm called “scVelo” [14], which includes a stochastic model and a dynamical model in addition to the steady-state model. The stochastic model treats the transcription, splicing and degradation as probabilistic events, which means steady-state levels are approximated not only from mRNA levels, but also from intrinsic expression variability. The dynamical model considers non-stationary populations and different splicing rates across genes, and the dynamics is solved in a maximum likelihood framework using the expectation maximization (EM) algorithm. The dynamical model is slower but can provide more consistent velocity estimation and better identification of transcriptional states.

The concept of RNA velocity and its associated algorithms and models have become very popular in single-cell biology. However, this technique needs the support of RNA sequencing protocols. Moreover, to get splicing information we need to run a complex preprocessing pipeline which involves the issues of file format and is time consuming. More importantly, the data of estimated RNA velocities are still sparse compared with the size of the uncovered cell state space. Here we propose an ensemble learning pipeline for the prediction of RNA velocities, which can skip the complex procedures for splicing analysis, etc. When we have a new data sample from the same biological context, we can predict the direction of RNA velocity from a state unkown in the traning data. This is similar to the pedestrian prediction [15] in a driverless transportation system, or the prediction of next movements of basketball players on the court [16]. It is possible to further combine all the transient movements into long trajectories of cells. Inspired by the concept of Waddington’s epigenetic landscape, which is a classical metaphor for cell differentiation, we can treat cells as balls rolling down through a potential surface. Based on the predicted RNA velocities and cell trajectories, we can reconstruct the landscape, as an intuitive platform for single-cell data visualization.

## Methods

### Velocity estimation

Velocyto CLI or loompy/kallisto was used to obtain spliced/unspliced reads annotations. We filter the genes with counts number (both spliced and unspliced) smaller than the threshold, keep the top high variability genes. Then normalize in cell level and did logarithm transform. On Euclidean distances PCA space of counts matrix, a nearest neighbor graph was computed, first and second moments were obtained for each cell. According to the basic reaction kinetics:

1 $$\begin{aligned} \frac{dU(t)}{dt}= & {} \alpha _{k}(t) - \beta \cdot U(t) , \end{aligned}$$

2 $$\begin{aligned} \frac{dS(t)}{dt}= & {} \beta \cdot U(t) - \gamma \cdot S(t) , \end{aligned}$$

where *S(t)* represents mature mRNA abundance over time, *U(t)* represents pre-mRNA abundance over time, $\alpha$ is the rate of transcription, $\beta$ is the rate of splicing, and $\gamma$ is the rate of degradation. *k* and *t* are cell-specific latent variables, where *k* represents discrete transcriptional state, and *t* represents latent time.

RNA velocity is termed as the time derivative of mature spliced mRNA $v(t) = \frac{dS(t)}{dt}$. Three approaches are provided in scVelo to do velocity estimation: steady state model, stochastic model and dynamical model. The basic difference between them is that the assumptions about the parameters are different. The data preprocessing steps are shown in Algorithm 1. For the sake of completeness and readers’ convenience, we have rephrased their description of methods for RNA velocity estimation into the pseudocode. After velocity estimation we can get a multi-dimensional RNA velocity vector *V* for each transcriptional state of a single cell. Combining this information we can further inference cell future state of an individual cell. The movements can be UMAP projected into a lower dimensional embedding *D* to visualize.

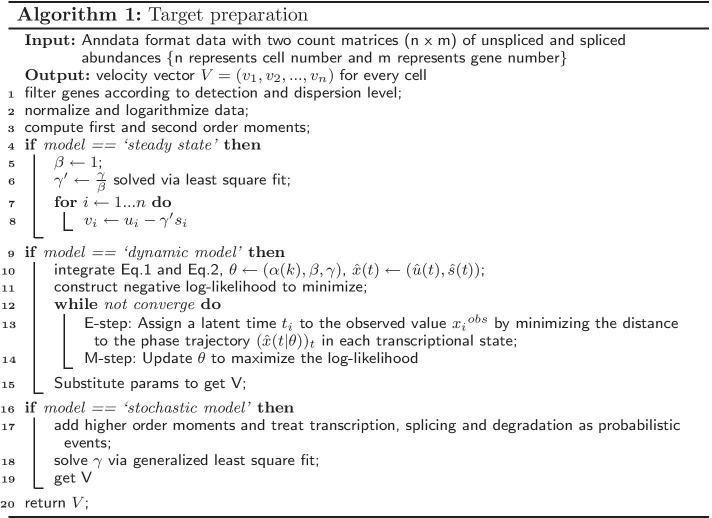



### Problem statement

**Fig. 1:**
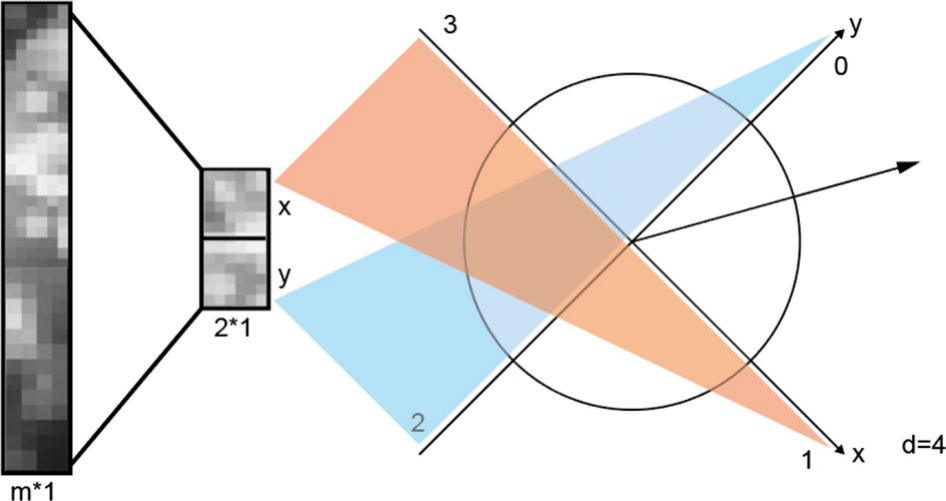
Circle partition. Assign *d* classification labels through equal division of a two dimensional circular plane. Here $d=4$

Our goal is to predict the RNA velocity vector of each cell based on its gene expression data. As illustrated in a 2D space, we formulate it as a classification problem through an equal division of a 2D circle into d equal-sized segments, as shown in Fig. 1. If the predicted and the original target directions fall in the same segment, we count it as a true positive, etc. A slightly more realistic formulation of the problem could be the regression of angles of the RNA velocities from a fixed direction. But we will leave that as a future work.

### Input preprocessing

**Fig. 2:**
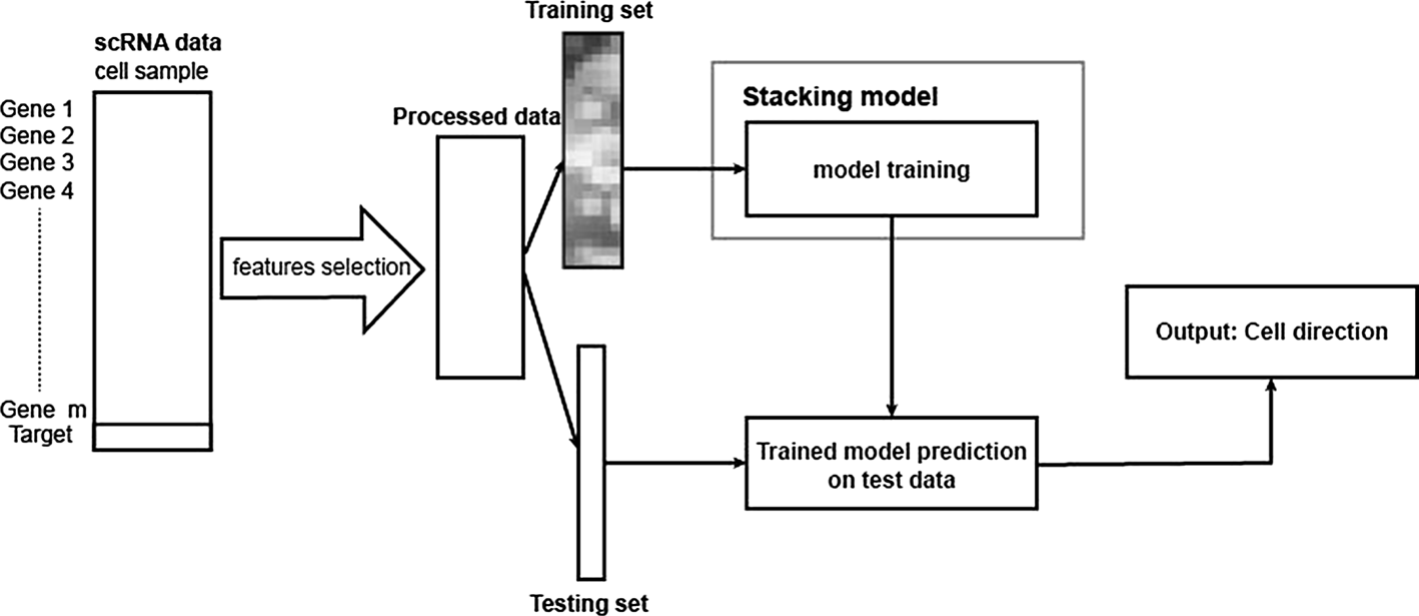
Overall pipeline of Velo-Predictor. The pipeline includes stages of feature selection, model training and prediction

Figure 2 shows the whole pipeline of our work. We start our supervised learning task from the gene expression matrix. Single-cell genomic data may be sparse and suffer from technical noise and bias. Therefore, to improve the behavior we need to do a de-noising step, or called feature engineering step. There are several ways to do such as scVI [17], scVAE [18] and DCA [19]. They basically use auto-encoder to find the hidden layer with minimum reconstruction error. After comparison we do feature selection based on gene ranking by ScVelo. ScVelo ranking is based on cluster-specific t-test to find genes with significantly higher/lower differential velocity, and we select the top *k* genes from each cluster as the model features. We use the lower-dimensional embedding $D\in \mathbf {R}^{2n}$ obtained from the velocity estimation step as our ground truth.

**Fig. 3:**
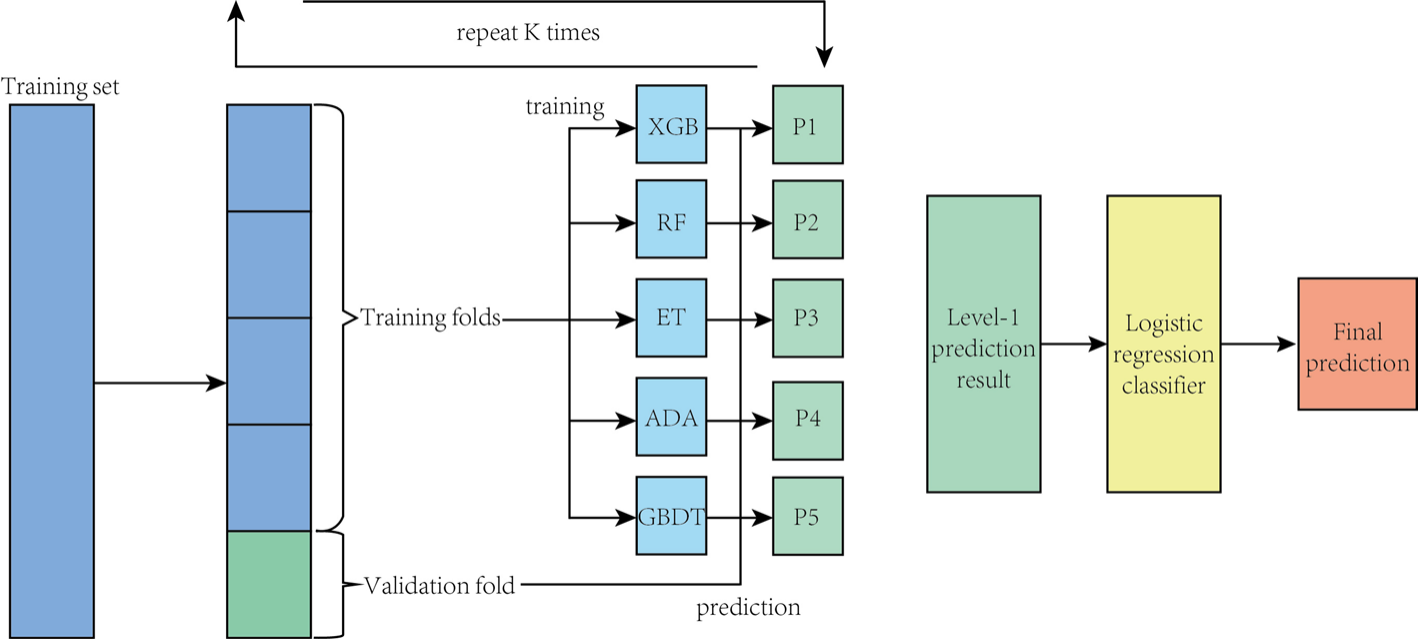
Architecture of the stacking model. Stacking with K-fold cross validation, where the first layer includes XGBoost, Random Forest, etc. as the base models. The second layer (as the meta classifier) is logistic regression classifier

After the division, the distribution of labels is not balanced. Therefore, we provide several ways to rescue: over-sampling, down-sampling and combine-sampling. We first test different sampling ways on different base models. For oversampling, we test adaptive synthetic (ADASYN) [20], synthetic minority oversampling technique (SMOTE) [21] and some variants of SMOTE, such as border line smote (BLS) [22] and svm-smote which uses support vector machine (SVM) [23]. For down-sampling, cluster centroid (CC) [24], random under sampler (RUS), NearMiss [25], repeated edited nearest neighbours (RENN) [26], neighbourhood cleaning rule (NCR) [27] and one side selection (OSS) [28] are used. Then, we further test combine-sampling methods of SMOTETomek [29] and SMOTEENN [30].

### Model training

We divide the sample data into a training set and test set. The training set is for model training and test set is for model evaluation. For training, the parameters are saved and can be directly used for prediction in testing part. We adapt a stacking structure model. Figure 3 and Algorithm 2 show the detail. We use mlxtend [31] and Scikit-learn [32] packages for implementation. We choose random forest (RF), GBDT, extra tree classifier (ET), adaboost (ADA) and XGBoost model to be the first layer, and the second layer is a simple Logistic regression classifier. To avoid over-fitting, we use cross validation concept to divide the training set to *K* subsets, where $K-1$ subsets are used to fit the first layer of classifiers. Then in each round, the unused subset will be predicted by the fitted classifier, and all the resulting predictions are stacked to feed into the second layer.

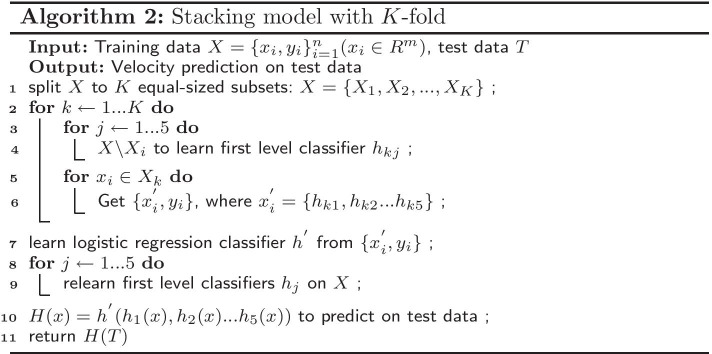



## Results

### Data sets

We train and test the models on two single-cell RNA-seq datasets. One is the Mouse hippocampal dentate gyrus neurogenesis (DGN) dataset [33] available from NCBI Gene Expression Omnibus (GEO) under accession ID GSE95753. It consists of RNA-seq data of 13,913 genes and  2930 cells from multiple lineages. The other dataset is Pancreatic endocrinogenesis (PE) [34] also available from NCBI GEO under accession ID GSE132188, which comprises the transcriptional levels of 27,998 genes of 3,696 pancreatic epithelial and Ngn3-Venus fusion cells sampled from mouse embryonic day 15.5. The number of cells and the numbers of genes (with different values of *k*, the number of top genes selected from each cluster as features) are shown in Table 1.

**Table 1:** Data statistics

	DGN	PE
Cell number	2930	3696
Gene number	13,913	27,998
Gene number (top k = 3)	41	24
Gene number (top k = 5)	63	38

**Fig. 4:**
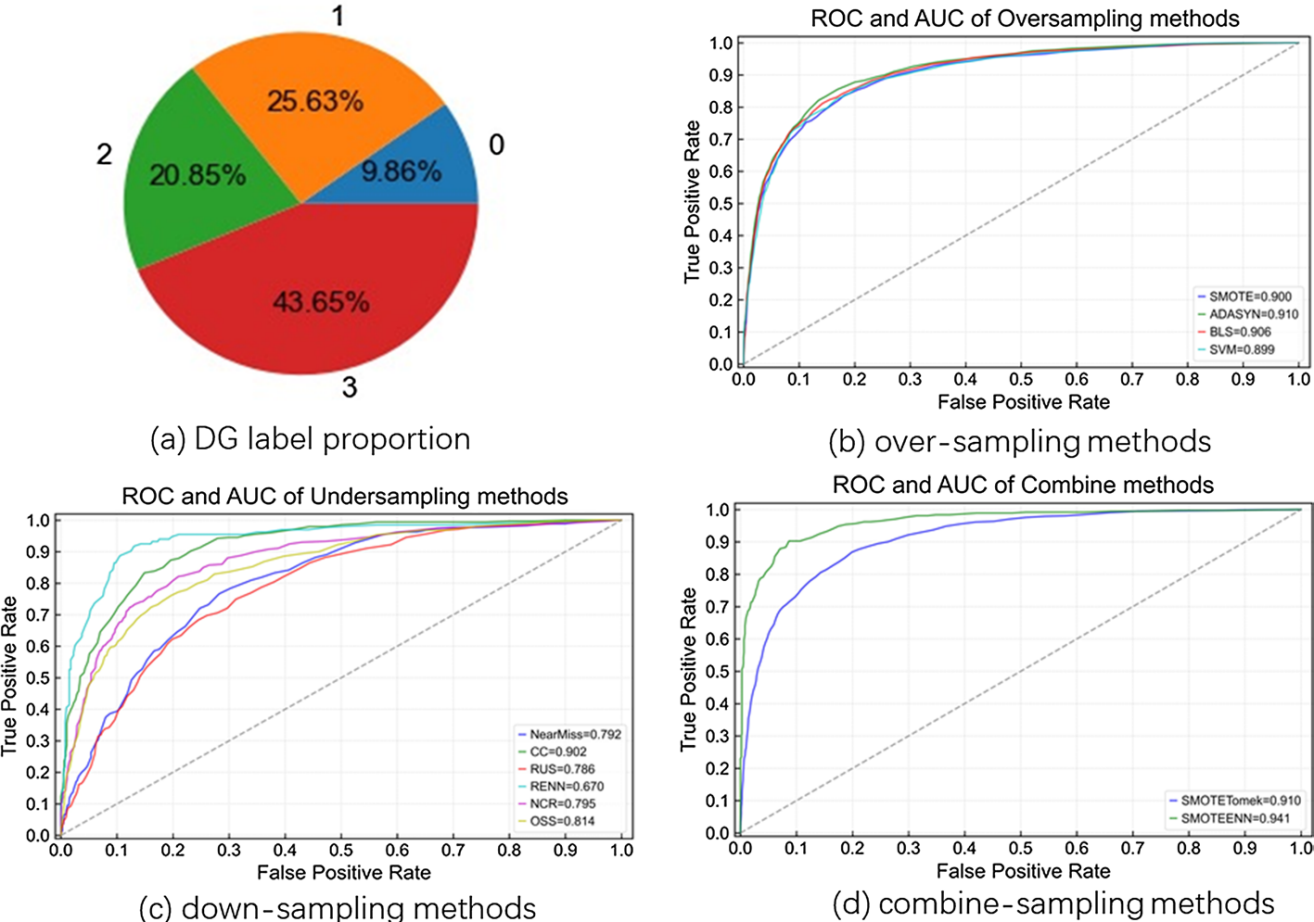
Methods comparison. **a** Direction label proportion of the DG dataset. **b**–**d** Performance of different methods to solve the class imbalance issue on the DG dataset

To test the generalization ability of our models, we randomly divide the cell samples into two disjoint sets with ratio of 7:3, 7 for training and 3 for testing. Figure 4a shows the proportion of labels in the DG datasets.

### Class imbalance issue

To illustrate how to address the class imbalance issue, we take the DG dataset as an example. Figure 4a shows the label proportion of the DG dataset. Figure 4b–d shows the ROC curve and corresponding AUC score of different sampling strategies. The AUC score is not enough for imbalanced data, thus we also consider the precision on each of the four classes and the balanced score as metrics. The metrics can be calculated according to the following equations:

3 $$\begin{aligned}&PRE(precision) = \frac{TP}{TP+FP} \\&Balanced\,Score = \frac{TPR+TNR}{2} \\ \end{aligned}$$

Table 2 shows the most representative performance of each methods. We can see the down-sampling methods perform poorly because of loss of information. The over-sampling methods are better but may introduce some biases. The best way is to combine them. Therefore we choose SMOTETomek as our final choice for the DG dataset.

**Table 2:** Performance of Random Forest on the DG dataset with different sampling methods

	PRE-0	PRE-1	PRE-2	PRE-3	Balanced score
Origin	0.65	0.68	0.57	0.66	0.52
SMOTE	0.8	0.75	0.69	0.67	0.73
NCR	0.67	0.72	0.67	0.75	0.55
ADASYN	0.8	0.76	0.7	0.68	0.73
NearMiss	0.67	0.61	0.46	0.46	0.53
SMOTETomek	0.8	0.77	0.71	0.7	0.75
SMOTEENN	0.83	0.81	0.74	0.4	0.64

### Functional analysis

**Fig. 5:**
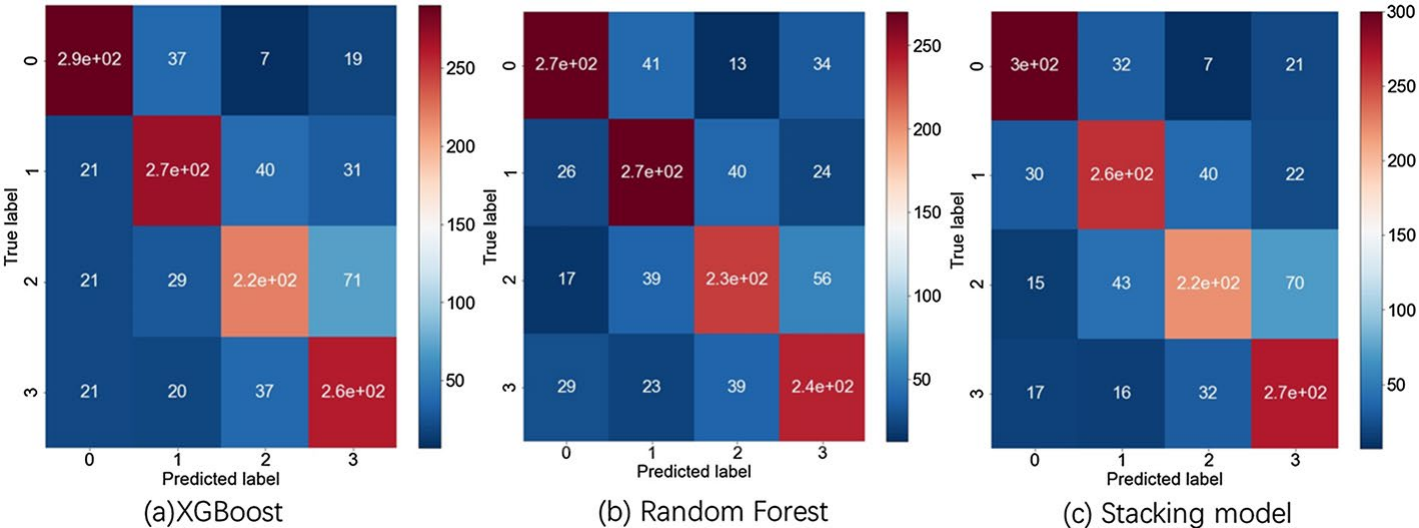
Confusion matrix of different models. Here shows the case when the number of classes *d* equals 4 on the DG dataset. **a** The performance of the XGBoost model, where each square corresponds to a predicted label and a true label for direction of RNA velocity, and the value in each square is the number of cells meeting the condition (color becomes darker as the number increases). We can see that the diagnals have darker colors which means that some samples are correctly classified. **b** The performance of the Random Forest model. **c** The performance of the Stacking model

**Fig. 6:**
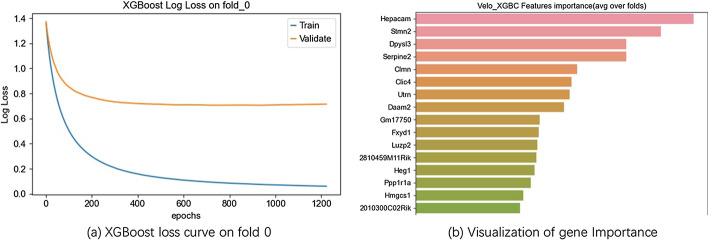
Loss curve and most important genes of XGBoost. **a** Training and validation log loss curve along epoches of XGBoost on DG dataset. **b** Gene importance ranking of XGBoost

**Fig. 7:**
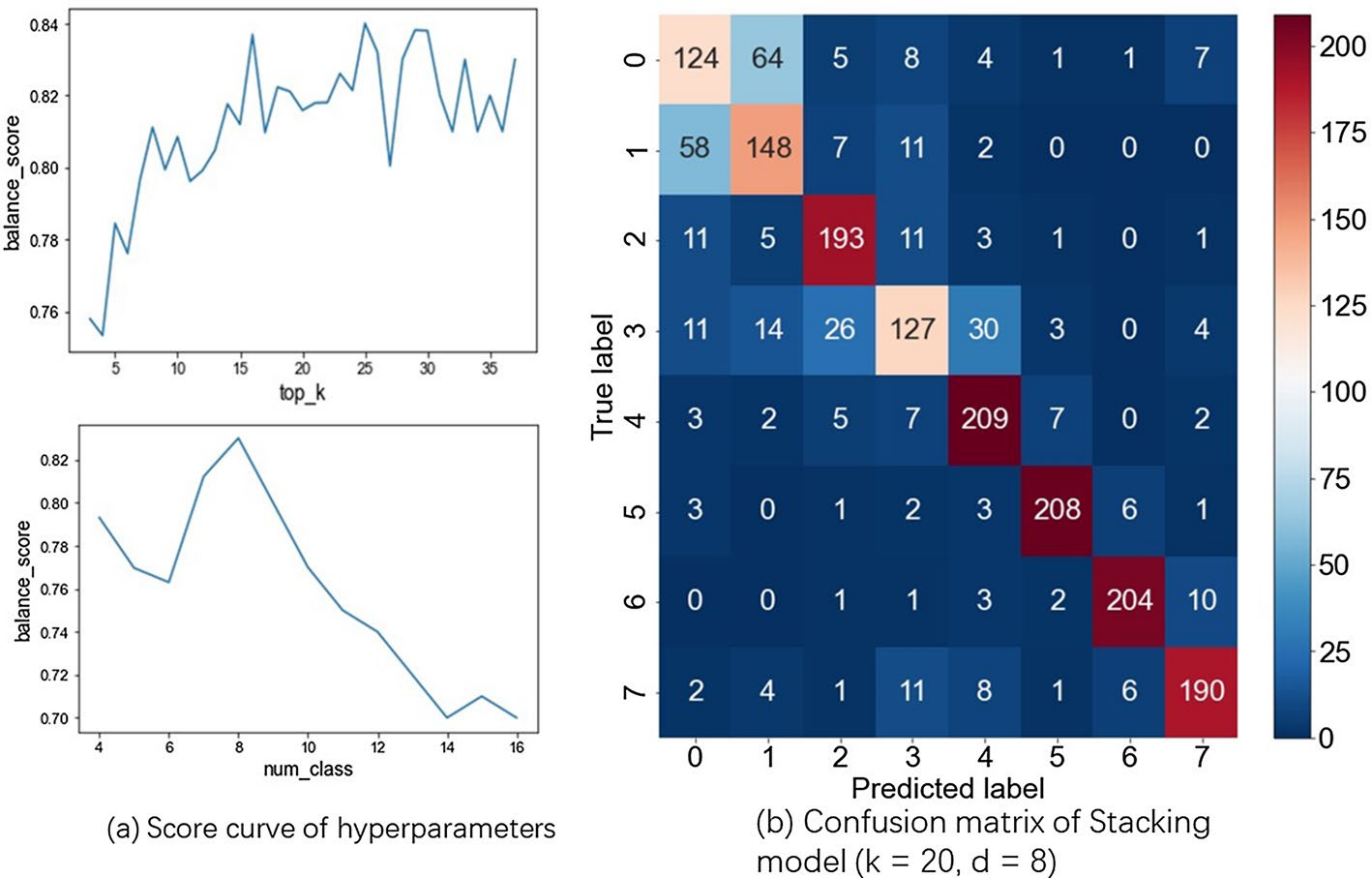
The impact of hyperparameters on DG dataset. **a** Balance score curve with different hyperparameters. The upper one shows the balance score increases with the increasing top gene number k. After k reaches 20, the balance score begins to oscillate which means 20 is enough for a good performance. The bottom one shows the performance drops when the number of classes exceeds 8. **b** Confusion matrix of the Stacking model when the number of genes k equals 20 and the number of classes d equals to 8. Each square corresponds to predicted label and a true label, and the value in each square is the number of cells satisfying the condition (color is darker as the number increases). The numbers along the diagnal show the performance of the Stacking model on DG dataset

After parameters fine tuning through grid search, Fig. 5 visualizes the performances of base models and stacking model. Figure 6a shows the loss curve on first fold, the behaviors of the other folds are similar. XGBoost model can also provide the log loss curve and the most important genes learning from the data (Fig. 6b). The impact of hyper parameters *k* the number of top genes and *d* the number classes is shown in Fig. 7b. Parameter *k* controls the feature selection part. The curve rises first and after *k* reaches 20, it starts to oscillate. In the previous experiment we set *k* to 3, although we can increase *k* to get better performance. Parameter *d* controls the granularity of prediction, and the result shows that the number of divisions is 8. When we continue to increase *d*, the task becomes more difficult so that the score will decay. Figure 7a shows the best performance of stacking model on DG dataset with $d = 8$, $k = 20$. Table 3 shows the comparison of base models and final stacking model. Stacking model’s performance is even slightly worse than XGBoost. We think that is probably due to the following reasons. First, the dataset is too small because stacking is not so strong when the data set is not big enough. Secondly, we can increase the model diversity of the first layer, and add more models to improve the performance. Thirdly, we did not use cross validation technique in the random forest model. In conclusion, we think the score difference is small and users can choose different provided models according to their size of data set.

**Table 3:** Performance of base and stacking models

	PRE-0	PRE-1	PRE-2	PRE-3	Balanced score
XGBoost	0.85	0.81	0.75	0.73	0.79
RF	0.83	0.8	0.72	0.66	0.75
GBDT	0.82	0.7	0.64	0.63	0.70
ET	0.87	0.79	0.72	0.73	0.76
ADA	0.66	0.58	0.55	0.58	0.60
stacking	0.83	0.8	0.74	0.7	0.78

### Visualization

**Fig. 8:**
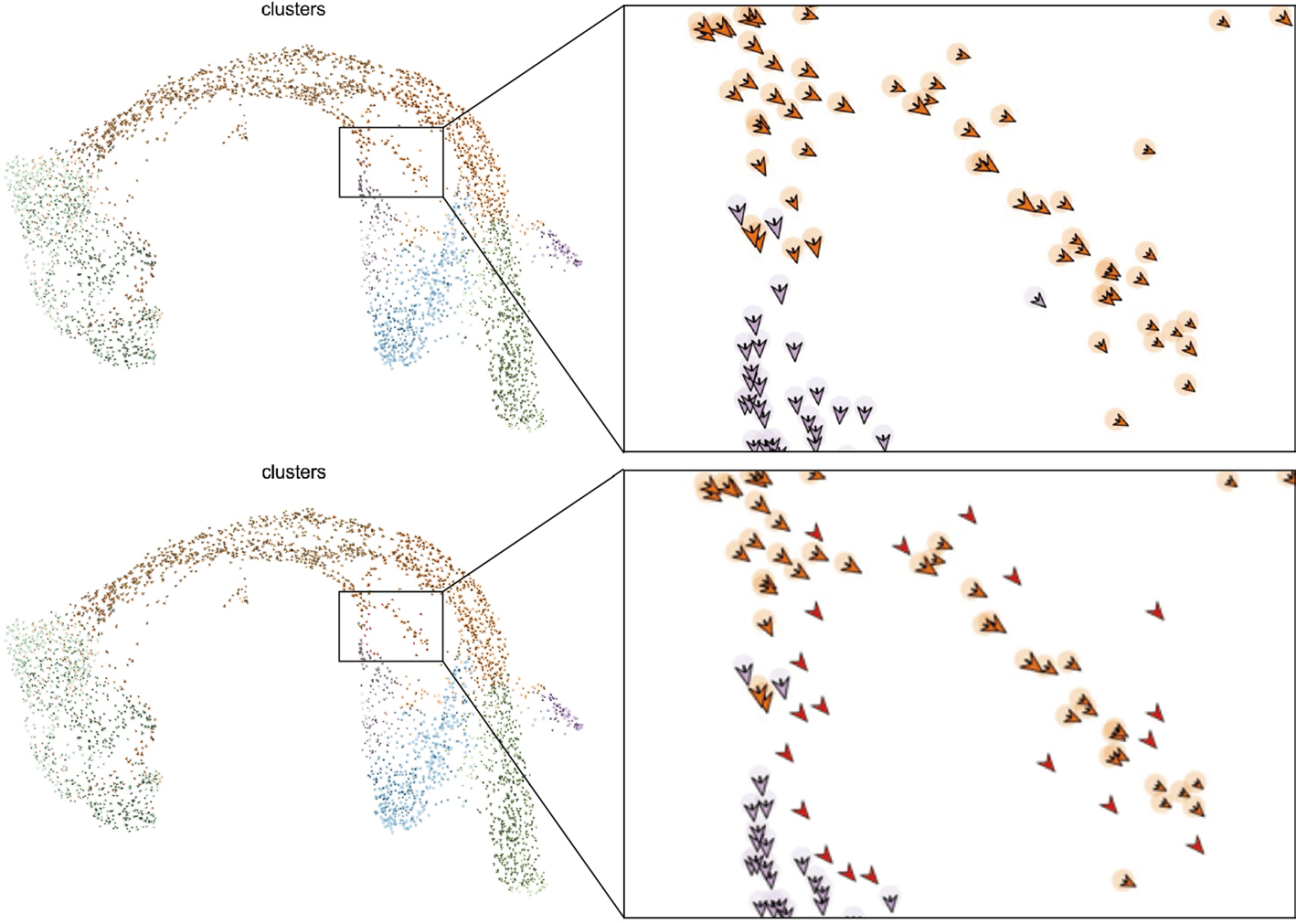
Schematic diagram of movement prediction on PE dataset. The leftside figure shows the velocity distribution of PE dataset, where different colors indicate different clusters. In the zoom-in figures on the right side, the upper one shows cells and velocity directions with original labels (training set), and in the bottom figure, the red arrows without halos represent cells and velocity directions with predicted labels (test set)

We use the UMAP toolkit of scVelo [14] to map cells and velocity vectors into two dimensional space, and we assume the incoming new data has the same distribution with our training data. We projected the new data in the same way as previous embedding, and give them a small red arrow which indicates the prediction of our velocity direction information. Each point in the figure represents a cell, the arrow is the velocity information of cells. It gives us an intuitive instruction of which way where a specific cell goes to. In Biology, it will tell us the differential path of a cell, we can see it performs well. Figure 8 shows the result on dataset PE, different colors indicate different clusters, above figure is the ground truth. In below figure, red arrow is our prediction outcome. Comparing with the same location in ground truth figure, we can see that the outcome is consistent with the ground truth. For detail, the orange dots represent pre-endocrine cells, and the perple dots represent epsilon cells. Through the zoom-in window, the comparision shows clearly that cell movements on 2D space are well captured.

## Discussion

One limitation of Velo-Predictor is that its performance may depend on data. Although we have used the ensemble learning framework to balance the results from different baseline models for different sample-feature ratios, it is still an empirical approach. When the data distribution is not so complete and balanced, the prediction may be less accurate. The lowest input data size depends on the properties of data (e.g. in terms of samples and features) and biological scenarios (e.g. types of data). We have tested the case ($k = 5$, $d = 4$) on the DG dataset by adjusting the testing data size, and when it exceeds 40%, the number of errors will increase to hundreds. In general, more complete data that cover the dynamical processes in the biological scenarios under study would be much preferred. Besides, the interpretability of the model is still a challenge.

Our work provides a prediction-based approach for study of cell differentiation mechanisms. Such predictions can also help impute the state space not yet covered by the scRNA-seq data, and the interpolation can help construct a continuous landscape surface. In the future, we can use single-cell multi-omic data to learn the bifucation point of cell differentiation more precisely. With the imputed direction information we can further do trajectory inference. Combined with an energy function such as that in the Hopfield network model used in our previous work [8], the predicted RNA velocities can be used to model the Waddington’s epigenetic landscape. Therefore, the prediction of RNA velocity can give biologists an intuitive picture about the trend of cellular dynamics, which is informative for their research.

## Conclusion

In this paper, we described Velo-Predictor, an ensemble learning pipeline for RNA velocity prediction. While RNA velocity estimation is not straightforward, our pipeline can simplify the procedure by learning a predictive model from gene expression data. The results showed that our pipeline can predict the directions of cell state transitions accurately.

## Data Availability

The single cell RNAseq data are publicly available in Gene Expression Omnibus, accession numbers GSE95753 and GSE132188. The code is available at https://github.com/clay001/Velo-Predictor.

## Abbreviations

scRNA-seq: Single-cell RNA sequencing
EM: Expectation maximization
ADASYN: Adaptive synthetic
SMOTE: Synthetic minority over sampling technique
BLS: Border line smote
SVM: Support vector machine variant of smote
CC: Cluster centroid
RUS: Random under sampler
RENN: Repeated edited nearest neighbours
NCR: Neighbourhood cleaning rule
OSS: One side selection
RF: Random forest
GBDT: Gradient boosting decision tree
ET: Extra tree classifier
ADA: Adaboost
DGN: Dentate gyrus neurogenesis
GEO: Gene Expression Omnibus
PE: Pancreatic endocrinogenesis
